# Path Loss Investigation in Hall Environment at Centimeter and Millimeter-Wave Bands

**DOI:** 10.3390/s22176593

**Published:** 2022-08-31

**Authors:** Md Abdus Samad, Dong-You Choi, Kwonhue Choi

**Affiliations:** 1 Department of Information and Communication Engineering, Yeungnam University, Gyeongsan-si 38541, Korea; 2 Department of Electronics and Telecommunication Engineering, International Islamic University Chittagong, Chittagong 4318, Bangladesh; 3 Department of Information and Communication Engineering, Chosun University, Gwangju 61452, Korea

**Keywords:** 5G, hall, indoor, interference, path loss, wave propagation, wireless communication

## Abstract

The millimeter-wave (mmWave) frequency is considered a viable radio wave band for fifth-generation (5G) mobile networks, owing to its ability to access a vast spectrum of resources. However, mmWave suffers from undesirable characteristics such as increased attenuation during transmission. Therefore, a well-fitted path loss model to a specific environment can help manage optimal power delivery in the receiver and optimal transmitter power in the transmitter in the mmWave band. This study investigates large-scale path loss models in a university hall environment with a real-measured path loss dataset using directional horn antennas in co-polarization (H–H) and tracking antenna systems (TAS) in line-of-sight (LOS) circumstances between the transmitter and receptor at mmWave and centimeter-level bands. Although the centimeter-level band is used in certain industrialized nations, path loss characteristics in a university hall environment have not been well-examined. Consequently, this study aims to bridge this research gap. The results of this study indicate that, in general, the large-scale floating-intercept (FI) model gives a satisfactory performance in fitting the path loss both in the center and wall side links.

## 1. Introduction

During the next decade, mobile data traffic is expected to increase 1000-fold, which will requiring wireless transmission techniques to be upgraded for the Fourth Industrial Revolution [1]. To facilitate higher data transmission capacities, along with other factors, the currently used 3 GHz frequency band must be moved to an higher band because cellular networks mostly run under 3 GHz, and most frequency bands below 3 GHz are already in use. Furthermore, the efficiency of the air interface spectrum is reaching capacity [2]. Therefore, there is a need to explore the wireless transmission characteristics of higher frequency bands for wireless links in 5G and 6G networks. However, they suffer from significant propagation loss because the diffusion losses from partitions and obstructions are higher in these extended-frequency bands [3]. Consequently, accurate path-loss assessment techniques are required for these bands in different environments. It is important to characterize these propagation effects when designing and implementing next-generation 5G and 6G radio-link networks. Proper design can be used to anticipate coverage, develop cellular networks, and manage the power levels of radio links. To construct accurate channel models in the expected higher frequency ranges, many academic and business ventures worldwide have made significant observations over the past several years.

In addition, path loss models are also valuable for determining localization in indoor environments where the conventional global positioning system (GPS) does not work due to signal blockage [4]. In [5,6], indoor localization systems were implemented using received signal strength (RSS), where the RSS was modeled using the path loss prediction technique. Similarly, a path loss model can help develop an RSS-based sensing mechanism for a localization system inside the auditorium.

Various wave propagation techniques have been investigated and published in this subject area. These include numerical techniques for solving electromagnetic wave propagation [7], ray tracing technique [8,9], modal technique [10], scaling technique [11,12], neural network [13], and empirical method [14]. The vector parabolic equation [15] and the finite difference time domain technique [16] have also been used to calculate the propagation of electromagnetic waves while reducing the computational complexity. In recent years, a combination of the two strategies has been proposed in many studies to minimize the computational load [17,18].

In a confined space, ray tracing can be used to determine the received power of a radio communication link. However, it has several implementation and analytical drawbacks. For example, ray-tracing implementation that employs a delay-line time-domain analysis requires extensive bandwidth: a one-ns time delay requires a 1 GHz of bandwidth [8]. Ray tracing may also be implemented using 3D modeling, but this approach has difficulty achieving convergence, owing to the higher number of reflections [19].

Wave propagation methods can encounter multiple obstacles in closed settings, so such methods need to consider several factors (e.g., those of a closed-environment) and their effects on the path loss model, which leads to increased computational complexity. A large-scale path loss model can simply provide the overall path loss transmission behavior instead of relying on individual factors (such as modeling the wave propagation in a tunnel) [20]. The findings suggest that large-scale models might help simulate radio wave propagation within closed facilities. Most path-loss models account for the attenuation caused by factors obstructing radio-wave transmission. In recent years, large-scale attenuation parameters have been used to model radio wave propagation in interior spaces instead of addressing attenuation owing to individual elements [21,22]. Furthermore, long-term wave propagation modeling has been applied in public facilities, such as corridors [23], and railway tunnels [24]. Additionally, large-scale path-loss techniques have been deployed to determine path losses in hallways [25], and emergency exit facilities [26]. As such, we also applied large-scale techniques in this study to simulate the recorded path loss. We observed that the large-scale models met our expectations.

Another study [1] experimented with wave propagation in an indoor hall scenario at 26 GHz. They studied several propagation parameters, such as the average power delay profile, root-mean-square delay spread, channel gain, and the K factor. In general, channel measurements and characterizations are required to explain wave propagation (and path loss) frameworks for various frequencies and environments. One such example is the characterization of wave propagation in an indoor hall. Although propagation research for 5G coexistence with mmWave frequencies has been ongoing in many distinctly relevant frequency bands and conditions, the characterization of the 5G channel model in each environment has yet to be investigated. According to the literature, infrastructure influences the propagation of radio waves. Consequently, researchers have become interested in finding models for radio wave propagation in settings such as residential areas [27,28], and streets [29]. Other indoor locations include offices [30], classrooms and laboratories [31], and shopping malls [32]. Contributions in [33] in-depth analyses and comparisons of the work in this field have been done by several organizations.

The operational frequency band of the 5G network is 3.5 GHz in Korea. It’s close to the 3.7 GHz band we used in the measurement campaign to avoid operational frequency interference with existing 5G network users in the neighborhood area. Meanwhile, 28 GHz frequency bands are expected to be allocated in both South Korea [34], and the United States [23] in the near future. Thus, this study investigated the path hall environment through large-scale modeling with operational frequencies of 3.7 and 28 GHz. We set up an experiment to analyze the path losses in an indoor hall environment at centimeter (3.7 GHz) and millimeter (28 GHz) frequency bands with horn-horn and horn-tracking antenna system (TAS) antennas that had not previously been studied.

Some of our contributions are as follows:

- We observed the wave propagation in a university hall environment and determined the path losses by placing the transmitter and receiver inside the hall.
- We used a horn and TAS-type antenna system and measured the path loss by tilting the antenna 15${}^{{}^{\circ}}$.
- The recorded received power was used to determine the optimized environment and frequency-dependent specifications of large-scale techniques, e.g., close-in (CI), floating intercept (FI), CI with frequency-weighted loss component (CIF), and the alpha-beta-gamma (ABG) model.

The remainder of this paper is structured as follows: Section 2 provides the experiment-specific situations and detailed explanations of their associated parameters. A description of the large-scale models is presented in Section 3, and Section 4 includes visual illustrations of the simulated large-scale path loss models, generated path losses, and measured data. The results obtained from the experiments are discussed. The conclusions are discussed in Section 5.

## 2. Data Assessment Drive Equipment

This section describes the equipment used in the experiments: specifications of the devices used, a geometric description of the university hall, precautions taken during the measurement drive, and the measurement procedure. This section also discusses a preprocessing technique applied to the measured raw data.

### 2.1. Signal Generator and Vector Signal Analyzer

**Figure 1:**
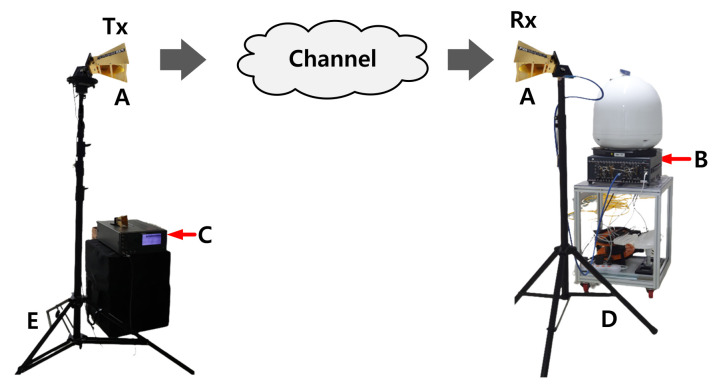
Channel sounder architecture. A—Double-ridged wave guide horn antenna, B—Keysight PXI 9393A signal analyzer, C—Keysight M5183B signal generator, D—AC power (immobile), Battery + inverter (mobile), E—AC power.

This section discusses the channel sounder and scenarios integrated into the measurement. The transmitting channel sounder was developed using a signal generator, cable connection, power supply, and horn antenna. The receiver channel consisted of a signal analyzer, cable connection, power supply, and antenna. We used a horn antenna with a $0^{{}^{\circ}}$ and $15^{{}^{\circ}}$ tilt and the TAS antenna system (Figure 1). The Keysight MXG N5183B and PXI 9393A devices were used for signal generation and analysis, respectively. The MXG N5183B is lightweight compared to other signal generators. It maintains a constant output power level and avoids overlapping spectra, which can disturb other frequency bands. The module can maintain a good noise level ≤−124dBc/Hz (10 kHz offset) with −75dBc spurious (at 10GHz). In addition, the signal generator offers easy calibration, with a switching rate of approximately 600μs. The collected signal was managed by a signal analyzer operating between 3.65 and 50 GHz. Additional specifications of the signal generator and vector signal analyzer are listed in Table 1 and Table 2, respectively. Additional parameters used in the experiments are listed in Table 3. Cable losses at the transmitter were 2.8 and 9.4 dB for the 3.7 and 28 GHz antennas, respectively. The cable losses at the receiver were 2 and 6.2 dB for the 3.7 and 28 GHz antennas, respectively.

**Table 1:** Specifications of signal generator.

Term	Specifications
Frequency	9 kHz–40 GHz
Resolution	0.001 Hz
Phase offset	0.01${}^{{}^{\circ}}$

**Table 2:** Specifications of vector signal analyzer.

Parameters	Specifications
Frequency range (GHz)	3.6–50
Analysis bandwidth (MHz)	40/100/160
Absolute amplitude accuracy (dB)	±0.13
Switching speed (µs)	<135
Displayed average noise level (dBm/Hz)	−168
Third-order intermodulation (dBm)	+31

**Table 3:** Channel specifications.

Parameters	3.7	28	28 (TAS)
Tx antenna type	Figure 2a	Figure 2b	Figure 2b
Rx antenna type	Figure 2a	Figure 2b	Figure 2c
Transmitter height (m)	1.75	1.75	1.75
Receiver height (m)	1.5	1.5	1.5
Tx antenna gain	10	20	20
Rx antenna gain	10	20	20
Overall gain (dB)	40	40	40
LNA gain	57	57	57
Polarization	Horizontal	Horizontal	Horizontal

**Figure 2:**
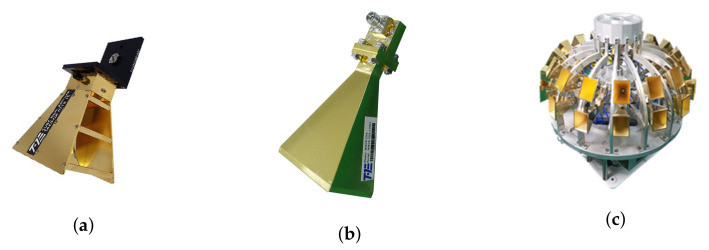
(**a**) TAEWA021810–double-ridged waveguide horn antenna used for 3.7 GHz operation. (**b**) WR2820A–Horn antenna used for 28 GHz. (**c**) TAS322640A–TAS antenna used for 28 GHz.

### 2.2. Properties of the Antenna

**Figure 3:**
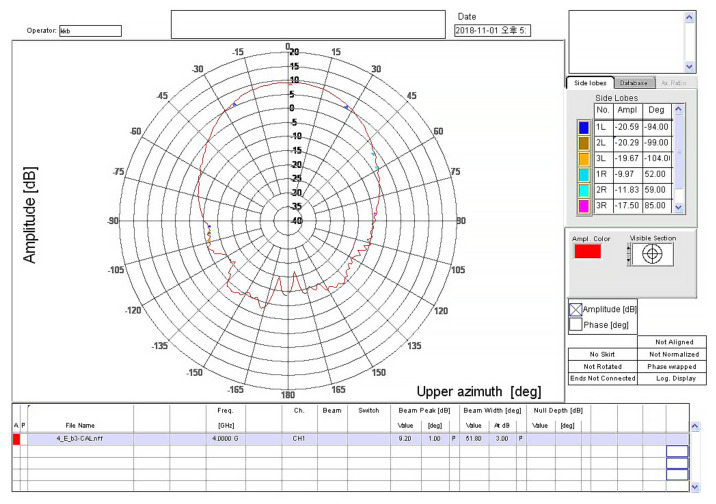
Gain of the double-ridged waveguide horn antenna at 4 GHz. The antenna documentation found that the same gain graph at 3.7 GHz was not readily available.

**Figure 4:**
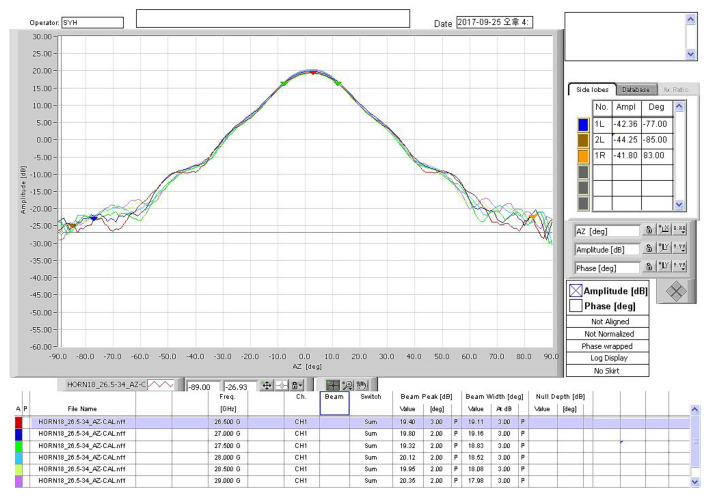
Horn antenna gain at 28 GHz.

**Figure 5:**
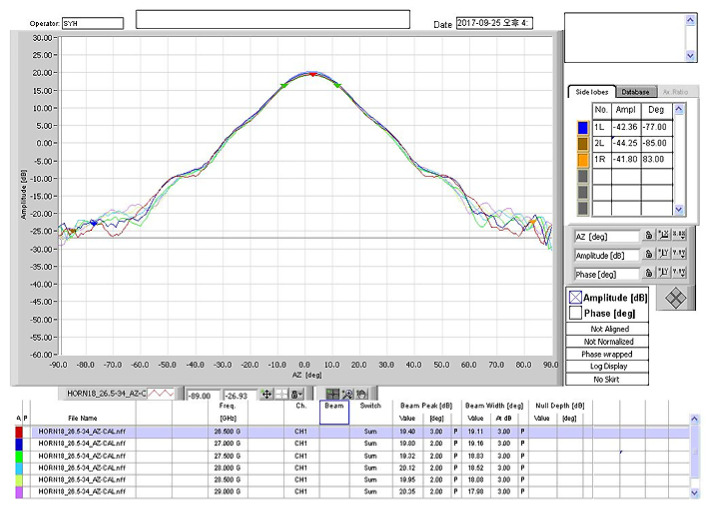
Gain of the TAS antenna at 28 GHz.

The TAEWA021810 and WR2820A horn antennas were used with operational frequencies of 3.7 and 28 GHz, respectively, as shown in Figure 2a,b. The frequency bands of antennas TAEWA021810 and WR2820A supported wide bands of 2–18 GHz and 26–40 GHz, respectively. Figure 2c shows the TAS antenna TAS322640A, which operated for 5G in the 25–40 GHz band. It required several functional blocks for collecting and analyzing signals from a field. The TAS antenna used a collection of waveguide horn antennas to receive power in all directions. It can use up to 32 antennas arranged in one to 16 horizontal and two vertical orientations. It also has a low-noise amplifier for working with the radio frequency front end and facilities for adjusting the height of the antenna system. To communicate with outside, the TAS antenna supports a GPS receiver, which may transmit information on a moving path and in a forward direction as data. The gain distributions of all antennas used in this study are shown in Figure 3, Figure 4 and Figure 5. In Figure 3, the gain from −90${}^{{}^{\circ}}$ to +90${}^{{}^{\circ}}$ is not readily available in the datasheet, whereas the gain plots in Figure 4 and Figure 5 are available in any direction.

### 2.3. Description of Data Assessment

#### 2.3.1. Structure of the Hall

**Figure 6:**
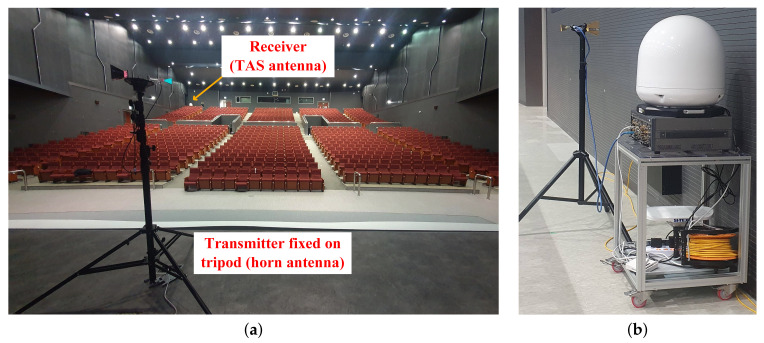
(**a**) Measurement scenario inside the hall of the Haeoreumgwan building at Chosun University, Korea. (**b**) Receiver.

**Figure 7:**
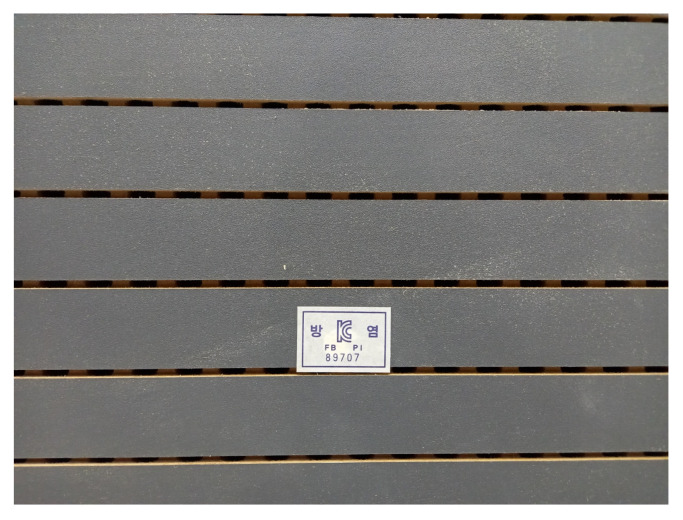
Inner side wall material.

**Figure 8:**
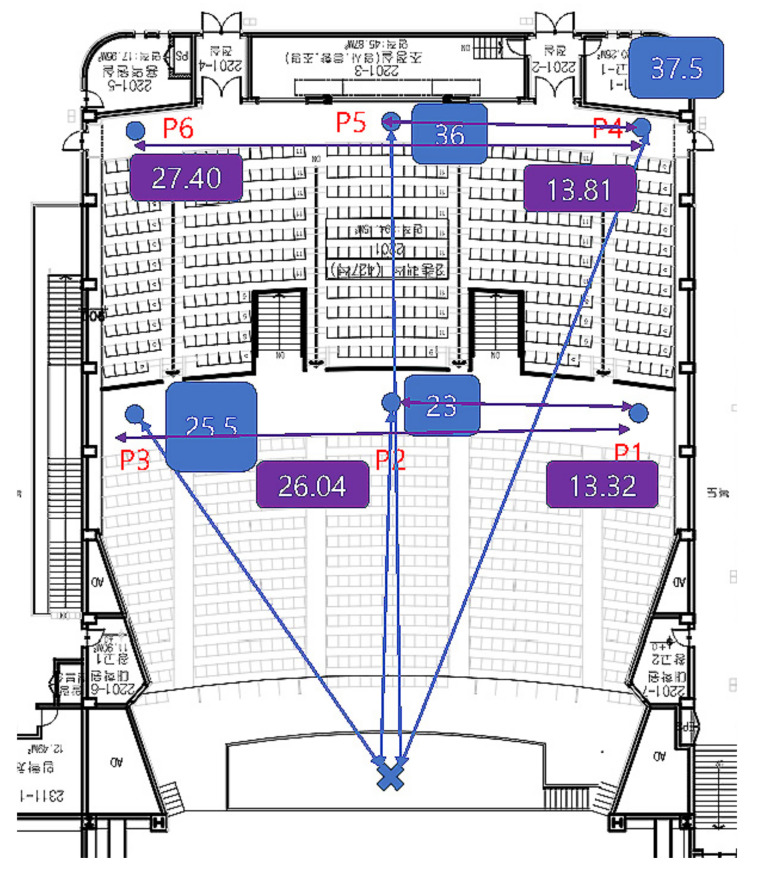
Theater layout. P_1_ = 26.57, P_2_ = 23, P_3_ = 25.5, P_4_ = 38.56, P_5_ = 36, and P_6_ = 38.48 m.

The channel measurement campaign was conducted in the main hall of Chosun University, South Korea, as shown in Figure 6. It has 990 seats and is considered the hall environment is a typical application scenario for 5G. All the seats were arranged into two tiers as shown in Figure 6. The distances between the transmitter and receiver varied, and the transmitting antenna was mounted on an adjustable tripod. Depending on the type of Rx antenna, it might be fixed on a tripod platform, whereas the TAS and omnidirectional antennas were placed on a mobile van. The transmitter and the receiver were mechanically supported on tripods at heights of 1.75 and 1.50 m, respectively. The hall was approximately 40 × 29.3 m, and the ceiling was different at different points. On the podium, the height was 6.9 m; between the aisle of first and second-tier seats, the height was 10.4 m; and in the last aisle of the second-tier seats, the height was 4.4 m. The ground, walls, and ceiling were concrete, but there were additional wooden structures on the inner wall, as shown in Figure 7. The seats were plastic and covered with fabric. The stage floor was made of wood. The wall at the center of the first-tier seats had an irregular structure, as shown in Figure 8. The auditorium had four metal doors—two on the front left and right side and two on the back left and right side.

No people or objects were included in the assessment during the measurements. The ceiling lights were switched on to prevent the room from being dark throughout the measurement, and the door was locked throughout. There was no additional staff in the hall during the measurements except for those who were usually there. During the measurement, no individuals were permitted to remain in the transmission area, for example, between the transmitter and receiver.

#### 2.3.2. Data Assessment Description

The transmitting antenna was mounted on a tripod positioned on the theater platform, and the receiving antenna was mounted on the mobile van. The transmitting and receiving antennas were kept 1.75 and 1.5 m above the floor level, respectively. The antenna gains for the 3.7 GHz horn antenna, 28 GHz horn antenna, and 28 GHz TAS antenna were 10, 20, and 20 dBi, respectively. The results from the study were compiled from data collected at six different locations, as shown in Figure 8. Table 4 explains the combination of the link and the antenna description. Points P_2_, and P_5_ are positioned in the theater’s center aisle, whereas points P_1_, P_3_, P_4_, and P_6_ are along the wall. We took several data points in these specific positions and considered the average.

In the datasheet, the measured path loss was not available. We obtained the measured received power at the receiver side from the vector signal analyzer. Therefore, we calculated the path loss per known loss and gain for the entire channel sounder. As all of the power is in the dB-scale, it was easy to compute the power loss in the wireless transmission channel by adding all of the gains (in the dB scale) and subtracting all the known losses in the system. Thus, the power loss in wireless transmission (*L*) can be computed as follows:

(1) $$L=\left(S_{1}+G_{2}+G_{3}\right)-\left(S_{2}+C_{1}+C_{2}\right)$$

In the above equation, $S_{1}$ is the transmitted signal power, $G_{2}$ and $G_{3}$ are the gains of the antennas used, respectively, for the transmitter and receiver, $S_{2}$ is the received signal power, $C_{1}$ is the cable loss at the transmitter, and $C_{2}$ is the receiver side cable loss.

## 3. Large-Scale Models

The technique used to determine the parameters for the FI, CI, CIF, and ABG models is discussed in the next section.

### 3.1. Propagation Technique with One Operational Frequency

#### 3.1.1. Close-in (CI) Model

The CI method provides an analysis of the large-scale channel oscillations resulting from the shadow effect [35]. If the parameter values are substituted into the following equation, the expected path loss for the CI technique can be written as follows [36]:

(2) $$\begin{array}{c} L^{CI}\left(f,d\right)=10n\log_{10}\left(d\right)+FL\left(f,1m\right)+X^{CI}\left[\mathrm{dB}\right];\;\mathrm{for}\;d\geq 1m \end{array}$$

Here, $X_{\sigma }^{CI}$ is a Gaussian random variable represented by the standard deviation (STD) $\sigma^{CI}$ and its mean is 0. FL(f,1m) is the path projected at a distance of 1 m from the transmitter source, and can be written alternatively as $10\log_{10}{\left(\frac{4\pi f}{c}\right)}^{2}$, and the symbol *n* shows the exponential factor in the path loss. It is possible to write (2) as follows:

(3) $$\begin{array}{c} X^{CI}=L^{\mathrm{CI}}\left(f,d\right)\left[\mathrm{dB}\right]-FL\left(f,1m\right)-10n\log_{10}\left(d\right);\;\mathrm{for}\;d\geq 1m \end{array}$$

If, for simplification, we assume that $A=L^{CI}\left(f,d\right)\left[\mathrm{dB}\right]-FL\left(f,1m\right)$, and $B=10\log_{10}\left(d\right)$, (2) becomes as follows:

(4) $$X^{CI}=A-nB$$

The STD of the shadowing factor (SF) can be defined as $\sqrt{\left\{\sum {\left(A-nB\right)}^{2}\;/\;L\right\}}$, where, *L* is the number of recorded different measurement data. Reducing the SF is equivalent to lowering the term $\sum {\left(A-nB\right)}^{2}$. Therefore, the first-order derivative $\sum {\left(A-nB\right)}^{2}$ with respect to *n* should be 0. Therefore, the value of the path loss exponent, *n*, may be determined using (5):

(5) $$n=\frac{\sum AB}{\sum B^{2}}$$

By determining the value of *n* from (5), the path loss of the CI model can be calculated using (2).

#### 3.1.2. Floating-Intercept (FI) Model

The FI method was used in the wireless world initiative new radio “WINNER) II” [37] and accepted for the 3rd generation partnership project (3GPP) standards of predicting path loss in wireless communication links [38]. The FI model of path loss is given by (6), as follows [36]:

(6) $$L^{FI}\left(d\right)\left[\mathrm{dB}\right]=\alpha +10\;\cdot \;\beta \log_{10}\left(d\right)+X^{FI}$$

Here, α indicates the intercepting parameter in dB unit, β denotes the slope of the line, and $X^{FI}$ is the Gaussian random variable with mean = 0 and STD = $\sigma^{FI}$.

The intercepting parameter (α) is equivalent to the free space path loss, and the slope (β) is equivalent to the PLE.

In (6), if we assume that $P=L^{FI}\left(d\right)\left[\mathrm{dB}\right]$, and $Q=10\log_{10}\left(d\right)$, we can write the calculation as follows:

(7) $$X^{FI}=P-\alpha -\beta Q$$

The STD of the SF can be written as follows:

(8) $$\begin{array}{c} \sigma^{FI}=\sqrt{\sum {\left(P-\alpha -\beta Q\right)}^{2}/L} \end{array}$$

As the term $\sigma^{FI}$ is supposed to vary the minimum, the term $\sum {\left(P-\alpha -\beta Q\right)}^{2}$ must be minimized, that is, its partial derivatives with respect to α and β must be equal to 0.

(9) $$\frac{\partial \sum {\left(P-\alpha -\beta Q\right)}^{2}}{\partial \alpha }=2\left(L\alpha +\beta \sum Q-\sum P\right)=0$$

(10) $$\frac{\partial \sum {\left(P-\alpha -\beta Q\right)}^{2}}{\partial \beta }=2\left(\alpha \sum L+\beta \sum Q^{2}-\sum PQ\right)=0$$

Equations (9) and (10) yield the following:

(11) $$\begin{array}{cc} \; & L\alpha +\beta \sum Q-\sum P=0 \end{array}$$

(12) $$\begin{array}{cc} \; & \alpha \sum Q+\beta \sum Q^{2}-\sum PQ=0 \end{array}$$

Combining Equations (11) and (12), we obtain the following.

(13) $$\alpha =\frac{\sum Q\sum QP-\sum Q^{2}\sum P}{{\left(\sum Q\right)}^{2}-L\sum Q^{2}}$$

(14) $$\beta =\frac{\sum Q\sum P-L\sum QP}{{\left(\sum Q\right)}^{2}-L\sum Q^{2}}$$

The best possible value for the STD of the SF can be computed by replacing the values of α and β with (7). Therefore, knowing α, β, and SF, the path loss can be calculated using (6).

### 3.2. Multi-Frequency Propagation

In [39], it was argued that a multi-frequency approach could be a helpful approximation for modeling path losses in indoor environments as there is a frequency-susceptible loss at a radius of 1 m in length around the transmitting source. This section discusses the “CIF” and the “alpha-beta-gamma” multi-frequency models for analyzing the experimentally obtained attenuation datasets.

#### 3.2.1. Close-in with a Frequency-Weighted Path Loss Exponent (CIF) Model

Modifications to the CI model allow for incorporating the frequency-dependent path loss exponent model–the CIF. The CIF uses the identical physical significance of the free space path loss at the radius of 1 m based on almost the same reason as the CI model.

(15) $$\begin{array}{c} L^{CIF}\left(f,d\right)\left[\mathrm{dB}\right]=FL\left(f,1m\right)+\left\{e\left(1-e\right)+\frac{eaf}{f_{0}}\right\}\;\cdot \;10\;\cdot \;\log\left(\frac{d}{1\;m}\right)+X^{CIF} \end{array}$$

In the above equation, *d* is a distance between the transmitter and the receiver larger than 1 m (unit in m), *e* is a PLE factor that represents the dependency of the attenuation while wireless transmission, $X^{CIF}$ is Gaussian random variable with a 0 mean and standard deviation σ(dB). *a* is the adjustment parameter used for the optimization that shows the attenuation slope owing to the frequency reliance. FL(f,1m) is the free space path projected at a distance of 1 m from the transmitter source and can be written alternatively as $10\log_{10}{\left(\frac{4\pi f}{c}\right)}^{2}$. *f*(GHz) is the carrier frequency, and $f_{0}$ is the marginal deployed frequency [40].

(16) $$f_{0}=\frac{\sum_{i=1}^{K}f_{i}N_{i}}{\sum_{i=1}^{K}N_{i}}$$

Here, *N* is the sum of the data logged in a unique frequency and antenna setup scenario, i∈K.

If we assume $R=L^{CIF}\left(f,d\right)\left[\mathrm{dB}\right]-FL\left(f,1m\right)$, $Z=10\log(\frac{d}{1m})$, x=e(1−a), and $y=\frac{ea}{f_{0}}$, from (15), we obtain as follows:

(17) $$X^{CIF}=R-S\left(x+yf\right)$$

The STD of the SF is as follows:

(18) $$\begin{array}{c} \sigma^{CIF}=\sqrt{\sum {\left\{R-S\left(x+yf\right)\right\}}^{2}/N} \end{array}$$

Lessening $\sigma^{CIF}$ is tantamount to $\sum {\left\{R-S\left(x+yf\right)\right\}}^{2}$. If the value of the term $\sum {\left\{R-S\left(x+yf\right)\right\}}^{2}$ is reduced to the minimum, its derivatives with respect to *x* and *y* should be 0, which leads to equations as follows:

(19) $$\frac{\partial \sum {\left\{R-S\left(x+yf\right)\right\}}^{2}}{\partial x}=2\left(x\sum S^{2}+y\sum S^{2}f-\sum SR\right)=0$$

(20) $$\frac{\partial \sum {\left\{R-Z\left(x+yf\right)\right\}}^{2}}{\partial y}=2\left(x\sum S^{2}f+y\sum S^{2}f^{2}\right.\left.-\sum SRf\right)=0$$

After simplification and combination, we obtain as follows:

(21) $$\begin{array}{cc} x & =\frac{\sum S^{2}f\sum SRf-\sum S^{2}f^{2}\sum SR}{{\left(\sum S^{2}f\right)}^{2}-\sum S^{2}\sum S^{2}f^{2}} \end{array}$$

(22) $$\begin{array}{cc} y & =\frac{\sum S^{2}f\sum SR-\sum S^{2}\sum SRf}{{\left(\sum S^{2}f\right)}^{2}-\sum S^{2}\sum S^{2}f^{2}} \end{array}$$

Equations (21) and (22), give the closed-loop solutions of the terms *x* and *y*, respectively. Simply substituting *x* and *y* into (18) gives the STD of the SF. Finally, the values of *e* and *a* can be determined by using the assumptions x=e(1−a) and $y=\frac{ea}{f_{0}}$, as given in Equations (23) and (24).

(23) $$e=x+f_{0}y$$

(24) $$a=\frac{f_{0}y}{x+f_{0}y}$$

Thus, by computing the value of *e*, *a*, and the SF (from (17)) the path loss can be calculated using (15).

#### 3.2.2. Alpha–Beta–Gamma (ABG) Model

The ABG model contains the parameter called α, β, and γ as tuning parameters to implement the frequency and distance-dependent variables. It uses a path loss model at the different frequencies [36]. The standard form of this model can be written as follows:

(25) $$\begin{array}{c} L^{ABG}\left(f,d\right)\left[\mathrm{dB}\right]=10\alpha \log_{10}\left(\frac{d}{1m}\right)+\beta +10\gamma \;\cdot \;\log_{10}\left(\frac{f}{1\;\mathrm{GHz}}\right)+X^{ABG} \end{array}$$

In the above equation, α is related to the path length, γ is associated with the frequency component of the link, β is applied as an offset having no physical significance. *f* is the operating frequency in GHz, and $X^{ABG}$ is a Gaussian random variable characterizing the deviations of the received power of the mean attenuation of the link.

The minimum mean square error optimization technique can determine the optimal values of factors α, β, and γ. The ABG method is applied to a given dataset by calculating the optimized values of the parameters α, β, and γ. To do so, let us assume that $U=L^{ABG}\left(f,d\right)\left[\mathrm{dB}\right]$, $V=10\log_{10}\left(d\right)$, and $W=10\log_{10}\left(f\right)$. Then (25) can be written as follows:

(26) $$X_{\sigma }^{ABG}=U-\alpha V-\beta -\gamma W$$

The STD of SF is given by (27) as follows:

(27) $$\begin{array}{c} \sigma^{ABG}=\sqrt{\sum {\left(U-\alpha V-\beta -\gamma W\right)}^{2}/L} \end{array}$$

As the slightest deviation is expected for the term $\sigma^{ABG}$, the such minimal deviation can be achieved through the partial derivatives of α,β, and γ and setting the whole term to 0 as follows:

(28) $$\frac{\partial \sum {\left(U-\alpha V-\beta -\gamma W\right)}^{2}}{\partial \alpha }=2\left(\alpha \sum V^{2}+\beta \sum V+\gamma \sum VW-\sum VU\right)=0$$

(29) $$\frac{\partial \sum {\left(A-\alpha L-\beta -\gamma R\right)}^{2}}{\partial \beta }=2\left(\alpha \sum V+L\beta \right.\left.\gamma \sum W\right.\left.-\sum U\right)=0$$

(30) $$\frac{\partial \sum {\left(U-\alpha V-\beta -\gamma W\right)}^{2}}{\partial \gamma }=2\left(\alpha \sum VW+\beta \sum W+\gamma \sum W^{2}-\sum WU\right)=0$$

From Equations (28)–(30), calculations can be derived as follows:

(31) $$\begin{array}{c} \alpha \sum V^{2}+\beta \sum V+\gamma \sum VW-\sum VU=0 \end{array}$$

(32) $$\begin{array}{c} \alpha \sum V+D\beta +\gamma \sum W-\sum U=0 \end{array}$$

(33) $$\begin{array}{c} \alpha \sum VW+\beta \sum W+\gamma \sum W^{2}-\sum W=0 \end{array}$$

By solving Equations (31)–(33), the path loss coefficients can be determined. Using (26) and the three coefficients α, β, and γ, the path loss can be calculated using the ABG model (25).

## 4. Results and Discussions

In the measured path losses dataset, we noticed links where the receiver was placed close to the wall (points P_1_, P_3_, P_4_, P_6_), suffered from higher path losses than those where the receiver was settled in the middle (points P_2_, P_5_) of the theater (see Figure 8). All of the five measurement cases—3.7 GHz antenna, 3.7 GHz $15^{{}^{\circ}}$ tilted antenna, 28 GHz antenna, 28 GHz $15^{{}^{\circ}}$ tilted antenna, and 28 GHz TAS antenna showed an average sudden higher received power at positions (points P_2_, P_5_) compared to their immediate previous position values (points P_1_, P_4_) by 4.9, 13.9, 23.8, 20.8, and 13.7 dB, respectively. The path loss model cannot be fitted with such abrupt high-variational datasets by a single model. Therefore, after separating middle side datasets, we built new wall side (WS) datasets consisting of the points P_1_, P_3_, P_4_, and P_6_ and center side (CS) datasets consisting of the points P_2_, P_5_. Consequently, we label two experimental locations, WS and CS. The received power was smooth after splitting the datasets into two parts compared to the previous unsplit datasets. Consequently, the measured path losses were modeled separately at the CS and WS positions.

**Figure 9:**
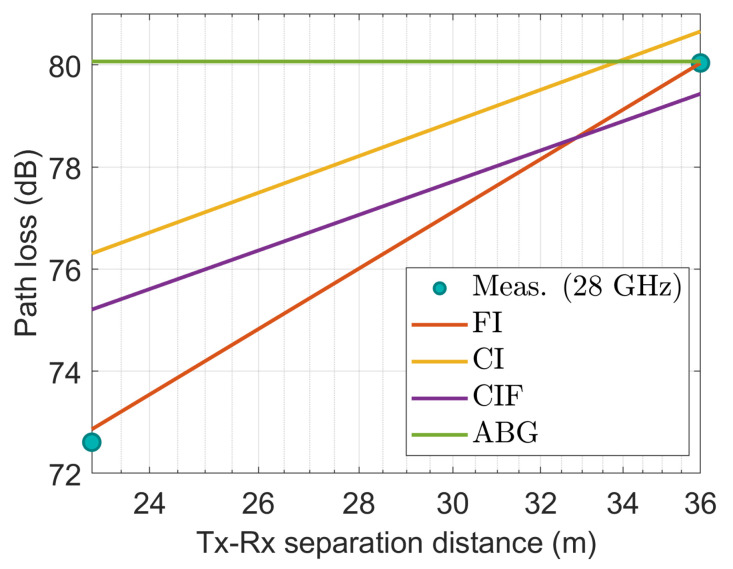
The measured data at CS positions modeled with large-scale models at 3.7 GHz.

**Figure 10:**
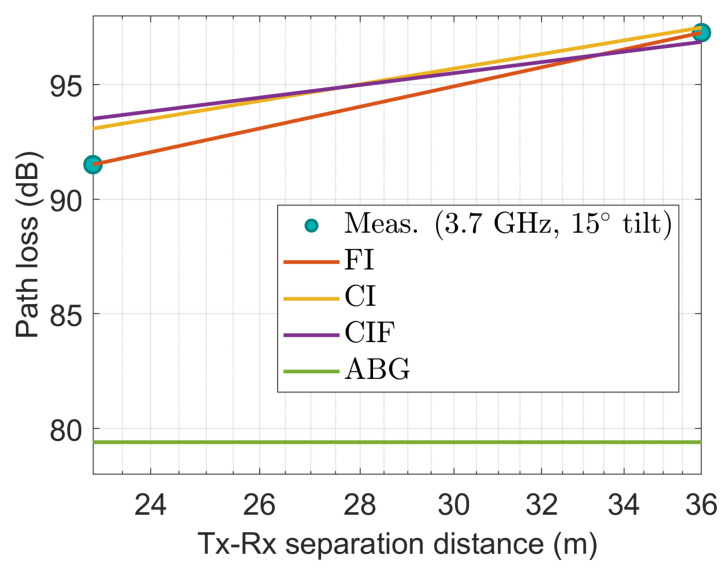
The measured data at CS positions modeled with large-scale models at 3.7 GHz with 15${}^{{}^{\circ}}$ tilted antenna.

**Figure 11:**
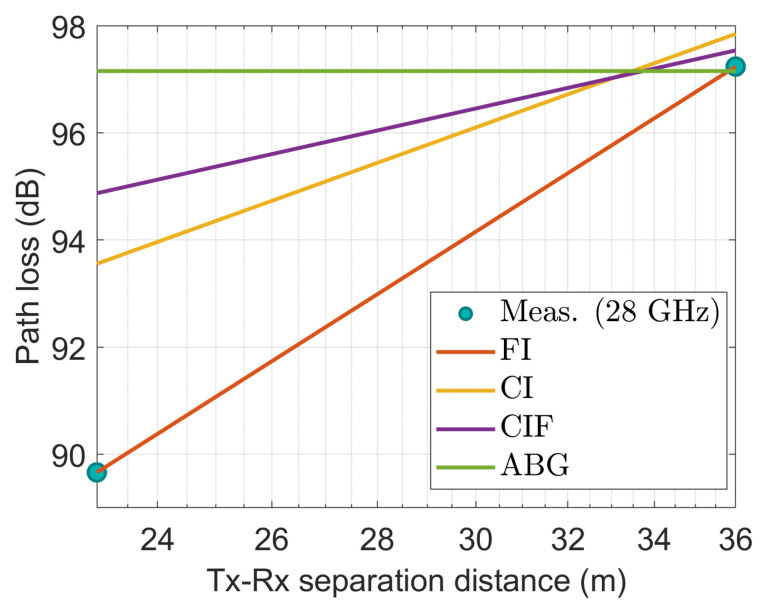
The measured data at CS positions modeled with large-scale models at 28 GHz.

**Figure 12:**
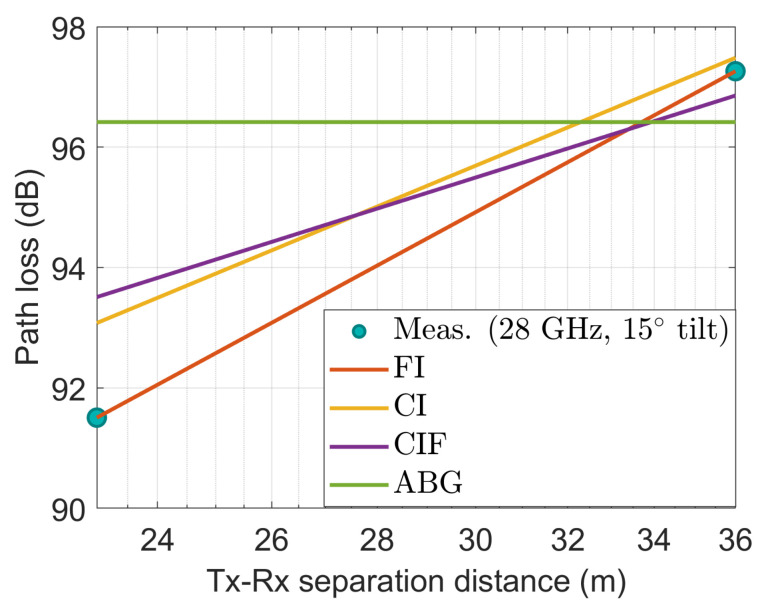
The measured data at CS positions modeled with large-scale models at 28 GHz using 15${}^{{}^{\circ}}$ tilted antenna.

**Figure 13:**
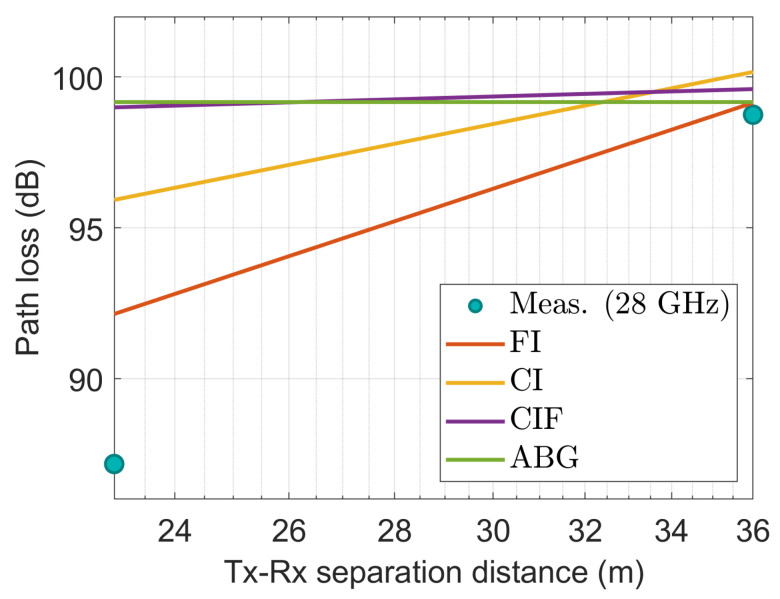
The measured data at CS positions modeled with large-scale models at 28 GHz using TAS antenna.

Figure 9, Figure 10, Figure 11, Figure 12 and Figure 13 show the measured path losses as fitted with the considered large-scale models (CI, FI, CIF, and ABG) with different antenna configurations and operational frequencies where the receiver antenna is placed in the middle of the auditorium.

Figure 9, the measured values correspond well to those of the FI model. The other three models–CI, CIF, and ABG show excessive path losses relative to the measured values, demonstrating their unfitness.

Figure 10 also shows that at 3.7 GHz with a 15${}^{{}^{\circ}}$ tilted antenna on the transmitter side, the measured values correspond well with the FI model. The performance of the CI and CIF model is also comparable to that of the FI model. However, the ABG model shows excessive depreciation relative to the measured values.

Figure 11 shows that the measured values agree well with the FI model at 28 GHz. The path loss prediction provided by the CI model is very close to the observed values at the far end but deviates by 4 dB at the near end. The CIF and ABG models exhibit more degraded prediction capabilities relative to the actual data.

It is seen in Figure 12 that the observed values agree well with the FI model at 28 GHz with 15${}^{{}^{\circ}}$ slanted antenna, and the CI and CIF model produce the second nearest path loss using the measured values. However, the ABG model significantly overestimates the data.

Figure 13 also shows that at 28 GHz with the TAS antenna, the measured values correspond best with those of the FI model among the CI, CIF, and ABG models. However, the CIF and ABG models produce excessive erroneous values compared with the physically measured path losses. In addition, the CI model does not provide an excellent fit for the data.

**Figure 14:**
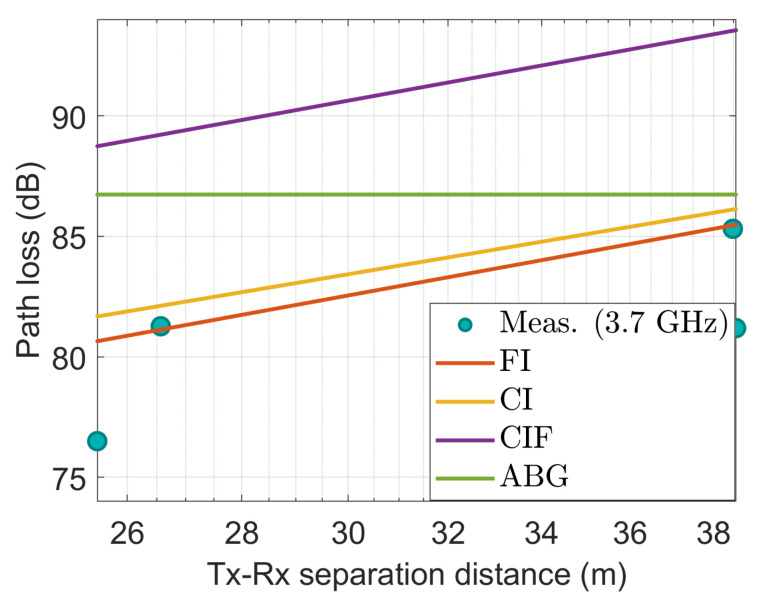
The measured data at WS positions modeled with large-scale models at 3.7 GHz.

**Figure 15:**
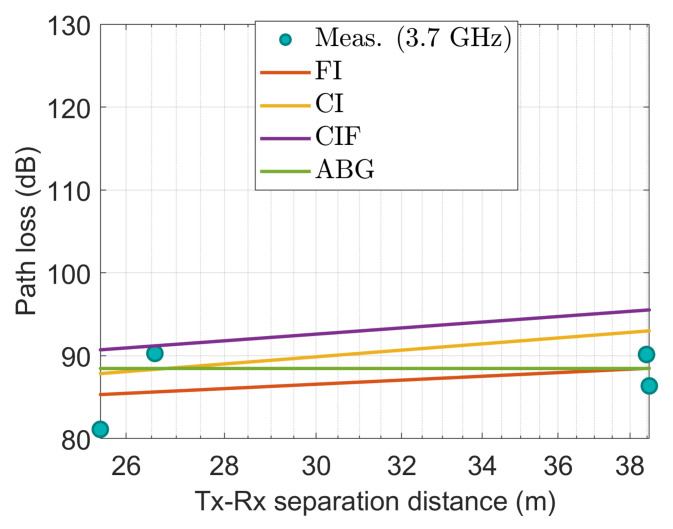
The measured data at WS positions modeled with large-scale models at 3.7 GHz using 15${}^{{}^{\circ}}$ tilted antenna.

**Figure 16:**
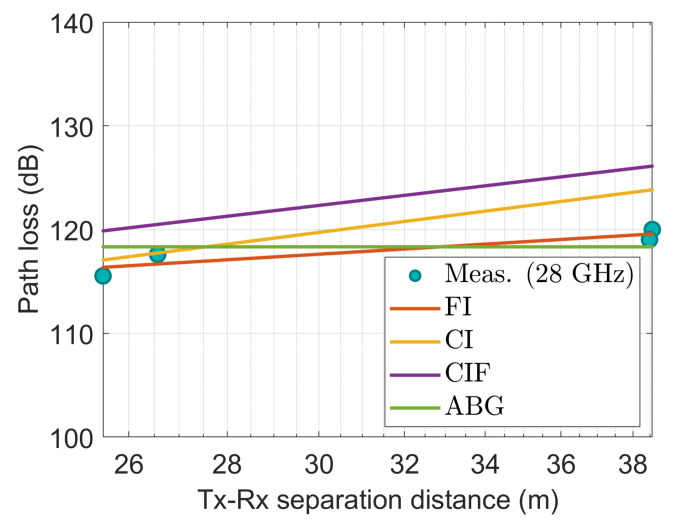
The measured data at WS positions modeled with large-scale models at 28 GHz.

**Figure 17:**
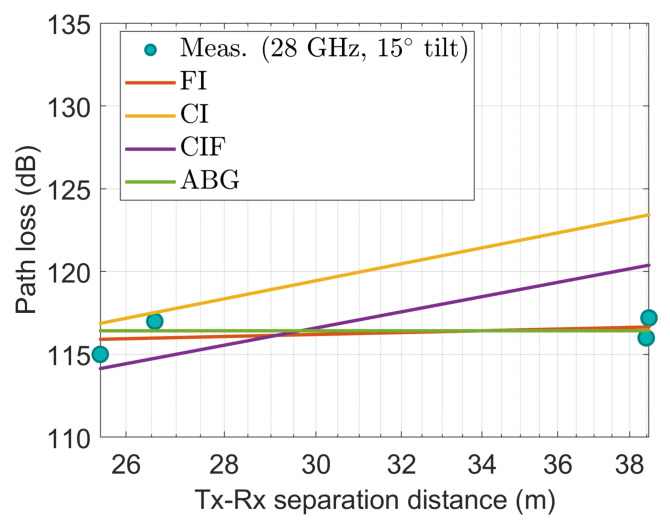
The measured data at WS positions modeled with large-scale models at 28 GHz using 15${}^{{}^{\circ}}$ tilted antenna.

**Figure 18:**
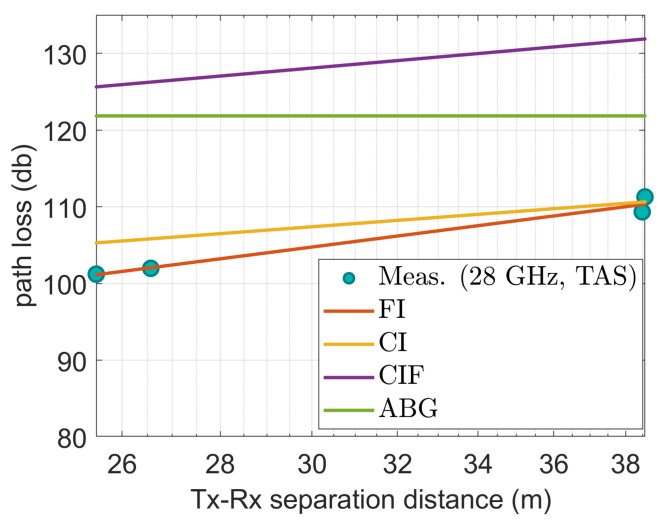
The measured data at WS positions modeled with large-scale models at 28 GHz using TAS antenna.

Figure 14, Figure 15, Figure 16, Figure 17 and Figure 18 show how the measured path losses match with large-scale models (CI, FI, CIF, and ABG) with different antenna configurations and operating frequencies when the receiver antenna is placed at the WS positions of the auditorium.

Figure 14 shows that when the FI and CI models are applied to a 3.7 GHz antenna, the observed values closely match the characteristics of the model. Despite this, the CIF and ABG models significantly overestimate the path loss compared with the experimental values.

Figure 15 also shows that the measured path loss at 3.7 GHz when the antenna was tilted at 15${}^{{}^{\circ}}$ does not fit well with the considered models. However, the CI, FI, and ABG models show almost identical performance. The CIF model shows deviated performance compared to the CI, FI, and ABG models.

Figure 16 also shows that the values measured at the 28 GHz antenna fit well with those of the FI and ABG models. The observed data do not fit well with the CI and CIF models.

The observed values at 28 GHz with a 15${}^{{}^{\circ}}$ slanted antenna also agree with the FI and ABG models as shown in Figure 17. In contrast, the CI and CIF models do not provide an excellent fit to the data.

Figure 18 also shows that at 3.7 GHz with a TAS antenna, the measured values correspond well to those of the FI model. The CI model is the second model that fits the measured data well at the far end, but it shows deviated performance at the near endpoint. The CIF and ABG models do not effectively match the observed data.

Table 5 and Table 6 show the statistical parameters developed by MMSE optimization in the CS and WS positions, respectively. The deviation of the predicted attenuation from the model can be estimated by shadowing factors in the Gaussian distribution. The shadowing factors in the CS and WS position areas are tabulated in Table 7 and Table 8, respectively. If we compare the effect of the shadowing factor at the CS and WS positions, generally, the shadowing factor increases (except for the 28 GHz TAS antenna configuration, in this case, the shadowing factor decreases from the CS to WS positions). In Table 7, we can see the general effect of the antenna tilt on the shadowing factor for the receiver location in the CS positions. If we compare the shadowing factor between the 3.7 GHz and 3.7 GHz $15^{{}^{\circ}}$ tilted antennas, the shadowing factor decreases for the four models in the CS positions. Similar behavior of the shadowing factor is observed for 28 GHz and 28 GHz $15^{{}^{\circ}}$ tilted antenna links in the CS positions. Similarly, in Table 8, the opposite trend of the effect of antenna tilt on the shadowing factor for the location of the receiver in the WS positions is observed. In the WS positions, the shadowing factor increases from 3.7 GHz ($0^{{}^{\circ}}$ tilt) to 3.7 GHz ($15^{{}^{\circ}}$ tilt) antenna links. In the same WS positions, the shadowing factor decreases from 28 GHz ($0^{{}^{\circ}}$ tilt) to 28 GHz ($15^{{}^{\circ}}$ tilt) antenna links for the CI and FI models. However, for the CIF and ABG models, the shadowing factor decreases from $0^{{}^{\circ}}$ to $15^{{}^{\circ}}$ tilted antenna.

## 5. Conclusions

In this paper, we have focused on comparing the performance of the CI, FI, CIF, and ABG path loss models in a university auditorium at frequencies of 3.7 and 28 GHz with previously unstudied antenna configurations. The results show that although the FI model predicts the path loss well, its parameters differ in the CS and WS locations. The results of this study underline that the FI model shows a match in a hall environment with a tilted antenna, an untilted antenna, and TAS antenna systems to locate the receiver antenna in the CS and WS positions. The ABG model offers a comparable performance limited to the specific WS positions (Figure 15, Figure 16 and Figure 17), except in the case where the shadowing factor is comparatively high, 8.78 (Table 8). The CI model shows a predominantly consistent performance next to the FI model. However, it never produced a good fit of the measured path loss, like in some cases, the good fitness capability of the ABG model. The CIF model generally did not show performance comparable to that of the other models. This work has revealed that the delivery of power to the receiver in a hall environment should consider that there is a significant difference in the power delivered to the CS positions compared to the WS positions. However, given the small sample size, caution must be exercised. Our results are encouraging and should be validated by a larger sample size. In our future work, we plan to develop offset parameters to generalize the FI path loss model at the CS and WS locations. We also plan to measure path loss in different auditorium sizes and compare the results.

## Figures and Tables

**Table 4:** Link configuration.

Transmitter	Receiver
3.7 GHz + Horn + 0${}^{{}^{\circ}}$ tilt	3.7 GHz + Horn + 0${}^{{}^{\circ}}$ tilt
3.7 GHz + Horn + 15${}^{{}^{\circ}}$ tilt	3.7 GHz + Horn + 0${}^{{}^{\circ}}$ tilt
28 GHz + Horn + 0${}^{{}^{\circ}}$ tilt	28 GHz + Horn + 0${}^{{}^{\circ}}$ tilt
28 GHz + Horn + 15${}^{{}^{\circ}}$ tilt	28 GHz + Horn + 0${}^{{}^{\circ}}$ tilt
28 GHz + Horn + 0${}^{{}^{\circ}}$ tilt	28 GHz + TAS + 0${}^{{}^{\circ}}$ tilt

**Table 5:** Path loss coefficients of CI, FI, CIF, and ABG models by setting receiver at the CS positions.

Freq. in GHz	CI	FI (α)	CIF	ABG (β)	CI (n)	FI (β)	CIF (n)	ABG (α)	CIF (b)	ABG (γ)
3.7	43.81	22.53	43.81		2.24	3.68				
3.7 (tilt $15^{{}^{\circ}}$)	43.81	38.02	43.81		2.10	2.05				
28	61.34	36.64	61.34	65.36	2.20	3.89	2.20	−0.00002	0.016	1.94
28 (tilt $15^{{}^{\circ}}$)	61.34	51.23	61.34		2.26	2.96				
28 (TAS)	61.34	40.80	61.34		2.18	3.60				

**Table 6:** Path loss coefficients of CI, FI, CIF, and ABG models by locating receiver at the WS positions.

Freq. in GHz	CI	FI (α)	CIF	ABG (β)	CI (n)	FI (β)	CIF (n)	ABG (α)	CIF (b)	ABG (γ)
3.7	43.810	40.68	43.81		2.48	2.69				
3.7 (tilt $15^{{}^{\circ}}$)	43.810	60.43	43.81		2.87	1.77				
28	61.34	91.16	61.34	63.49	3.77	1.79	3.16	−0.00010	0.19	3.44
28 (tilt $15^{{}^{\circ}}$)	61.34	110.09	61.34		3.64	0.41				
28 (TAS)	61.34	29.15	61.34		2.97	5.12				

**Table 7:** Shadowing factor of CI, FI, CIF, and ABG models by locating receiver at the CS positions.

Freq.	CI	FI	CIF	ABG
3.7	2.05	0.127	1.82	3.71
3.7 (tilt $15^{{}^{\circ}}$)	0.51	≈0	1.72	3.08
28	2.19	≈0	1.65	3.79
28 (tilt $15^{{}^{\circ}}$)	0.89	≈0	0.97	3.05
28 (TAS)	4.88	2.32	3.71	5.81

**Table 8:** Shadowing factoring of CI, FI, CIF, and ABG models by locating receiver at the WS positions.

Freq.	CI	FI	CIF	ABG
3.7	2.93	2.11	3.61	3.69
3.7 (tilt $15^{{}^{\circ}}$)	3.62	3.41	4.60	5.42
28	2.71	0.71	4.82	5.05
28 (tilt $15^{{}^{\circ}}$)	4.21	0.80	3.91	3.14
28 (TAS)	2.08	0.67	7.70	8.76

## Data Availability

Not applicable.
